# GOLM1 suppresses autophagy-mediated anti-tumor immunity in hepatocellular carcinoma

**DOI:** 10.1038/s41392-021-00673-6

**Published:** 2021-09-17

**Authors:** Tianqi Sui, Xiaoyang Wang, Lili Li, Junxiao Liu, Nan Qiao, Lihua Duan, Minxin Shi, Jianfei Huang, Heng Yang, Genhong Cheng

**Affiliations:** 1 Center for Systems Medicine, Institute of Basic Medical Sciences, Chinese Academy of Medical Sciences and Peking Union Medical College, Beijing, China; 2 Suzhou Institute of Systems Medicine, Suzhou, Jiangsu China; 3 Heze Vocational College, Heze, Shandong China; 4 Department of Rheumatology and Clinical Immunology, Jiangxi Provincial People’s Hospital, Nanchang, Jiangxi China; 5 Affiliated Tumour Hospital of Nantong University, Nantong Tumour Hospital, Nantong, Jiangsu China; 6 Department of Clinical Biobank, Affiliated Hospital of Nantong University, Nantong, Jiangsu China; 7 Department of Microbiology, Immunology and Molecular Genetics, University of California, Los Angeles, Los Angeles, CA USA

**Keywords:** Tumour immunology, Gastrointestinal cancer

**Dear Editor**,

Immune-mediated tumor elimination depends on the production of cytokines and the recruitment of immune cells in the tumor microenvironment. Cell death-related signals such as ATP release from tumor cells are crucial for the activation of downstream immune responses.^1^ GOLM1, also known as GOLPH2 and GP73 as a Golgi transmembrane protein involved in the transport of protein cargo through the Golgi apparatus has been extensively studied in various cancers for its multifunctional roles in promoting cancer proliferation and metastasis through the AKT/mTOR pathway.^2,3^ Its secreted form has been used as a serum biomarker in patients along with hepatocellular carcinoma (HCC) tumorigenesis and progression.^4^ However, its functional role in anticancer immunity is still unclear.

Our immunohistochemical results demonstrated that GOLM1expression levels were significantly higher in malignant HCC tissues than in benign liver tissues (Fig. 1a, b). Kaplan–Meier survival analysis indicated that HCC patients with high GOLM1 expression (cutoff value = 96.5) showed a worse prognostic factor (Fig. 1c). We further evaluated the inverse correlation between CD8 and GOLM1 expression levels in HCC tissues, which suggested that GOLM1 might be involved in immune regulation in HCC (Fig. 1d, supplementary Fig. S1a). Importantly, the tumors with a high expression level of GOLM1 were poorly differentiated, indicating that GOLM1 may predict the malignant progression of HCC (Supplementary Tables 1–3). Collectively, GOLM1 expression is elevated in tumor cells and inversely correlated with CD8^+^ T cell infiltration and clinical outcome in the tumor microenvironment of human HCC.

**Fig. 1:**
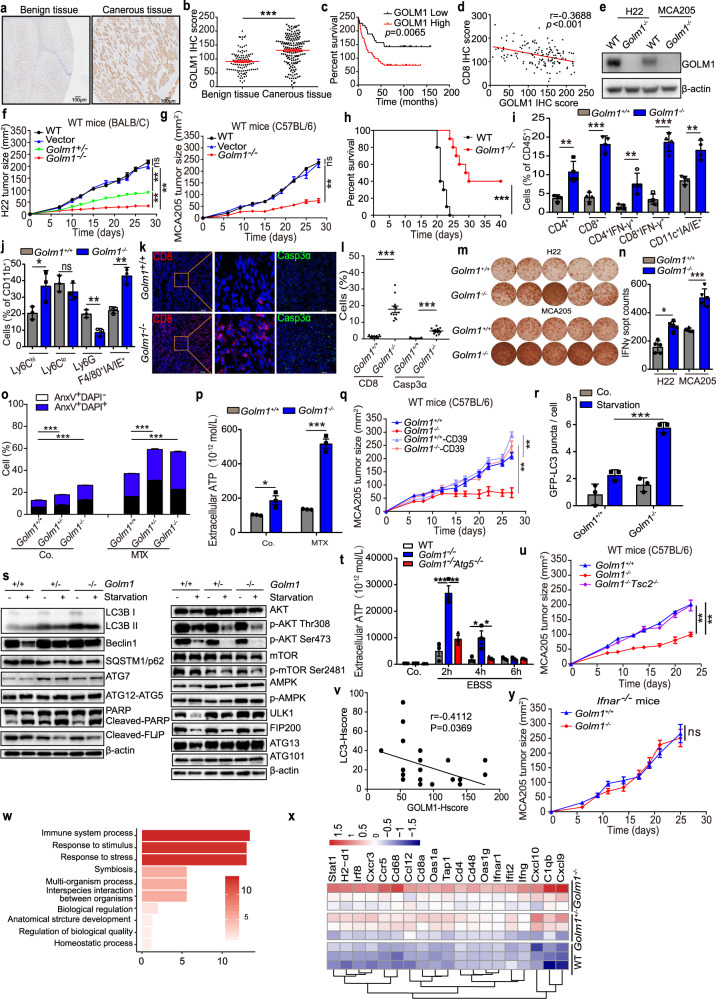
GOLM1 suppresses autophagy-mediated anti-tumor immunity in hepatocellular carcinoma. **a**, **b** Expression of GOLM1 immunohistochemical reaction in benign liver tissues (*n* = 48) and cancerous tissues (*n* = 161). Tan dyeing indicates positive GOLM1 staining in the cytoplasm of liver cells. **c** Kaplan-Meier cumulative survival curves of HCC patients grouped as low expression or high expression of GOLM1 (*n* = 101, cutoff value = 96.5). **d** Analysis of correlation between the expression of CD8 and GOLM1 in HCC tissues (*n* = 138). **e** Western blot by using an anti-mouse GOLM1 antibody showed successful gene knockout in H22 and MCA205 cells generated with a CRISPR vector carrying a scrambled guide RNA sequence. β-actin was used as a loading control. **f, g** Tumor growth curves of immunocompetent Balb/c mice subcutaneously inoculated H22 cells (**f**), and C57BL/6 mice inoculated MCA205 cells (**g**). Tumor progression is monitored 2-3 times per week and depicted as error bars of mean±SEM at each time point. Each group of tumor sizes contains 5 mice, and these results are representative of three independent experiments. **h** *Golm1*^−/−^ H22 and control cells were implanted into in mouse liver to establish an orthotopic HCC model and implanted mice survival was observed. **i**, **j** At the time point of 10 days, MCA205 tumors in wild-type C57BL/6 mice were harvested and processed to detect the indicated cell populations by flow cytometry. Total tumor-infiltrating lymphocytes (TILs) percentage in CD45^+^ cell population (**i**), and in CD11b^+^ cell population (**j**) were shown. **k, l** At the time point of 10 days, MCA205 tumors in wild-type C57BL/6 mice were harvested and processed to detect the indicated proteins by immunofluorescence microscopy. Immunofluorescence staining images of CD8 and Cleaved-Caspase3 were represented (**k**) and the expression level was summarized in **l** (Scale bars: 100 μm). **m**, **n** MCA205 tumors in wild-type C57BL/6 mice were harvested and processed to detect IFNγ secretion by ELISpot assay. **m** Representative images of ELISpot responses from H22 (up) and MCA205 (down) tumors. Each well is represented a mouse tumor, and the quantitative data are shown in **n**. **o** *Golm1*^*+/+*^, *Golm1*^*+/−*^, and *Golm1*^*−/−*^ H22 cells were treated with or without MTX for 24 h. Cell apoptosis was detected through staining with Annexin V plus vital dye DAPI followed by flow cytometry analysis and a quantitative summary is shown. **p** *Golm1*^*+/+*^ and *Golm1*^*−/−*^ H22 cells were treated with or without MTX for 24 h. Quantification of ATP secretion from cell supernatants immediately collected and detected by chemiluminiscence assay. **q** Growth curves of immunocompetent mice bearing *Golm1*^*+/+*^, *Golm1*^*−/−*^, CD39 overexpressing *Golm1*^*+/+*^ and CD39 over-expressing *Golm1*^*−/−*^ MCA205 tumors. **r** *Golm1*^*+/+*^ and *Golm1*^*−/−*^ MCA205 cell lines stably expressing RFP-GFP-LC3 reporter protein were generated via lentivirus-mediated overexpression. The cells that exhibited a large number of RFP-GFP-LC3 dots after treatment of EBSS (3 h) were analyzed by confocal immunofluorescence. Quantification of cells with Red-GFP-LC3 puncta is shown. **s** *Golm1*^*+/+*^, *Golm1*^*+/−*^, and *Golm1*^*−/−*^ H22 cells were treated with completed medium or Earle’s balanced salt solution (EBSS) for 3 h. Cell lysates were prepared to be available for western blot detection, the blots were exposed as indicated antibodies and further exposed to the respective secondary antibodies. Representative western blot analysis of autophagy-related proteins (left) and key components of AKT-mTOR signaling pathway (right). **t** WT, *Golm1*^*−/−*^ and *Golm1*^*−/−*^ *Atg5*^*−/−*^ H22 cells were treated with or without EBSS for the indicated time. Quantification of ATP secretion from cell supernatants immediately collected and detected by chemiluminiscence assay. **u** Growth curves of immunocompetent mice bearing *Golm1*^*+/+*^, *Golm1*^−/−^ and *Golm1*^−/−^ *Tsc2*^−/−^ MCA205 tumors. **v** Analysis of correlation between the expression of LC3 and GOLM1 in HCC tissues (*n* = 138, *r* = −0.3502, *P* = 0.0457). **w**, **x** *Golm1*^*+/+*^, *Golm1*^*+/−*^, and *Golm1*^*−/−*^ H22 tumors in Balb/c mice were isolated at day 10 after implantation, and gene expression was analyzed by RNA sequencing (*n* = 3/group). Top functional pathway items by GO analysis and IPA (**v**). Heat map demonstrates type I interferon-related genes with a P value of less than 0.05 and a fold change of greater than 2 over the control group (**w**). **y** Tumor growth curves of *Ifnar*^*-−/−*^ mice implanted with *Golm1*^*+/+*^ and *Golm1*^*−/−*^ MCA205 cells. The quantitative variables between the two groups are analyzed by the Mann–Whitney U test (**f**, **g**, **q**, **t**, **u**, **y**) or unpaired Student’s *t*-test (**b**, **i**, **j**, **l**, **n**, **o**, **p**, **r**). Correlation between two groups is by the two-tailed Pearson’s correlation analysis (**d**, **v**). Quantitative data are represented as mean ± SEM; ns, not significant, *P < 0.05, **P < 0.01, ***P < 0.001

To further determine the role of GOLM1 in anti-tumor immunity, we firstly generated several independent clones of H22 hepatoma and MCA205 fibrosarcoma cell lines that lacked *Golm1* expression (Fig. 1e, supplementary Fig. S1b). Both *Golm1*^−/−^ H22 hepatoma and MCA205 fibrosarcoma exhibited a vigorous reduction in tumor growth in immune-competent mice as compared with their WT parental cells (Fig. 1f, g). Interestingly, both *Golm1*^−/−^ H22 and MCA205 cells grew into tumors at similar sizes as their corresponding WT cells in T cell-deficient *nu/nu* mice (Supplementary Fig. S1c). Although abdominal massive malignant ascites produced in both groups at an early stage, the lack of *Golm1* expression in H22 tumor cells prolonged mice median survival significantly from 21 days to 29.5 days. In addition, 40% of mice have totally recovered at day 40 manifested by abdominal malignant ascites disappearance (Fig. 1h). These findings demonstrated that tumor regression associated with *Golm1* deficiency occurs in an immune-dependent fashion.

Indeed, the knockout of *Golm1* increased the number of tumor-infiltrating CD4^+^ or CD8^+^ T cells, IFNγ-production cytotoxic T lymphocytes, higher proportions of F4/80^+^ MHCII^+^ macrophages and CD11c^+^ MHCII^+^ dendritic cells including CD11b^+^Ly6C^hi^ cell subtype (Fig. 1i–l, supplementary Fig. S1d). Moreover, IFNγ secreted by TILs isolated from H22 and MCA205 *Golm1*^−/−^ tumors environment presented more than that of the corresponding *Golm1*^+/+^ tumors (Fig. 1m, n). Particularly, *Golm1*^−/−^ tumor displayed a greater proportion of apoptotic cells staining with the noteworthy expression of activated caspase-3 than in *Golm1*-sufficient controls (Fig. 1k, l), implying that increased cell death may trigger the recognition by antigen-presenting cell and elicitation of the specific antitumor immune response. Altogether, *Golm1* deficiency likely promotes T cells and APCs recruitment into tumors leading to increased production of cytokines like IFNγ.

Chemotherapy-induced immunogenic cell death (ICD) is expected to influence the composition and the architecture of tumor immune infiltration, which contributes to the elimination of residual tumor cells. In our study, Annexin V^+^DAPI^−^ subpopulations of *Golm1*^−/−^ cells were increased as compared to WT cells, suggesting that *Golm1* deficiency promoted the early stage of apoptosis (Fig. 1o, supplementary Fig. S1e). Consistently, the western blotting analysis indicated that the intracellular levels of cleaved-PARP and cleaved-Caspase8 (p43/41) were increased along with the reduction of cleaved-FLIP_L_ after MTX treatment in *Golm1*^−/−^ H22 cells (Supplementary Fig. S1f). More importantly, *Golm1* deficiency significantly increased the secretion of ATP in response to MTX treatment supporting that ATP might play a critical role in antitumor immunity in *Golm1*^−/−^ tumor (Fig. 1p). To abolish extracellular ATP in the tumor microenvironment, the ecto-ATPase CD39 was overexpressed on the surface of tumor cells. The presentence of CD39 on *Golm1*^−/−^ MCA205 cells significantly restored tumor growth to a similar rate as *Golm1*^+/+^ MCA205 (Fig. 1q). Thus, *Golm1* deficiency may promote antitumor immunity through an increased extracellular ATP release.

Selective autophagy helps to regulate the clearance of dying cells by the generation of energy-dependent engulfment signals including ‘eat me’ and ‘find me’ signals. We found that *Golm1* knockout increased the abundance of LC3 puncta and the expression levels of the key proteins of autophagy including LC3II/LC3I and ATG7 (Fig. 1r, s, and supplementary Fig. S2a). We observed that the autophagy upstream suppressors such as phospho-AKT (Thr308), phospho-AKT (Ser473), and phospho-mTOR (Ser2481) were reduced to lower levels in *Golm1*^−/−^ cells than *Golm1*^+/+^ cells after starvation. On the other hand, ULK1 complex proteins including ULK1, ATG13, and FIP200, which are essential to initiate autophagy, were maintained at high levels in *Golm1*^−/−^ cells instead of a strong reduction in *Golm1*^+/+^ cells under the starvation condition (Fig. 1s). To further verify the contribution of GOLM1 regulated autophagy formation to ATP release, we generated *Golm1*^−/−^ *Atg5*^−/−^ cells and observed that *Golm1*^−/−^ *Atg5*^−/−^ cells abolished the increased ATP release in *Golm1*^−/−^ cells (Fig. 1t). As TSC2 is an important autophagy activator in the ATK-mTOR signaling pathway, we have also generated *Golm1*^−/−^ *Tsc2*^−/−^ cells and observed that *Golm1*^−/−^ *Tsc2*^−/−^ tumors grew at similar rates as *Golm1*^+/+^ tumors, much faster than *Golm1*^−/−^ tumors (Fig. 1u). In addition, our HCC tissue microarray analysis indicated that the expression of GOLM1 is a negative correlation with the expression of LC3 (Fig. 1v). Together, the results further support that GOLM1 promotes tumor growth by suppressing autophagy formation and ATP release via the AKT/mTOR pathway.

RNA sequencing was performed to further explore the potential downstream molecules that may contribute to GOLM1 promoting tumor growth. Interestingly, the top three enriched Gene Ontology subsets were “immune system process”, “response to stimulus” and “response to stress” (Fig. 1w). Clustering analysis data indicated that the following categories of genes were upregulated in *Golm1* deficient cells as compared to *Golm1* WT cells: (1) chemokines and chemokine receptors like *Ccl12, Cxcl10, Ccr5*, and *Cxcr3*, which may be involved in the recruitment of T cells; (2) early myeloid genes like H2-d1 and Tap1 which were linked to antigen processing and presentation; (3) CD molecules like *Cd4, Cd8a*, and *Cd68*; and (4) IFN signaling or ISGs such as *Ifng, Stat1, Ifnar1, Oas1a, Ifit2*, and *Irf8* (Fig. 1x). We next compared the tumor growth rates between *Golm1*^−/−^ and *Golm1*^+/+^ cells in *Ifnar1*^−/−^ mice. As shown in Fig. 1y, the sizes of *Golm1*^−/−^ and *Golm1*^+/+^ tumors in *Ifnar1*^−/−^ mice were similar at multiple different time points. These results indicate the indispensable roles of the IFN pathway on *Golm1*^−/−^ tumor growth.

In summary, we found elevated GOLM1 in tumor cells correlated with reduced CD8 T cell infiltration into the tumor microenvironment and reduced prognosis of HCC in Chinese cohorts. More importantly, we have demonstrated that *Golm1*^+/+^ cancer cells grew tumors at a much faster rate than *Golm1*^−/−^ cancer cells in immune-competent mice but at a similar rate in immunodeficient mice, suggesting GOLM1 has a novel role in suppressing anti-cancer immunity.

Our studies have also provided evidence indicating that GOLM1 may inhibit immune response in the tumor microenvironment and promote tumor growth through potential mechanisms dependent upon the AKT/mTOR-mediated regulation of autophagy formation and extracellular ATP release (Supplementary Fig. S3). Interestingly, another paper found that GP73 upregulates PD-L1 expression by enhancing the level of EGFP and promoting the phosphorylation of STAT3, ^5^indicating that GP73 participates in anti-tumor immunity through kinds of pathways. So, future studies are required to further determine the molecular connections among these multiple different players. Nevertheless, our studies identified GOLM1 as a profound checkpoint blocker target for HCC immunotherapy.

## Supplementary information

Supplementary materials

## Data Availability

Data are available upon reasonable request.
