# Supplementary material for: GOLM1 suppresses autophagy-mediated anti-tumor immunity in hepatocellular carcinoma
Source: Signal Transduct Target Ther. 2021 Sep 17;6:335. doi: 10.1038/s41392-021-00673-6 (PMC8445956; doi:10.1038/s41392-021-00673-6)
Supplement: Supplementary file 1 — Supplementary materials [file 41392_2021_673_MOESM1_ESM.docx]

Supplementary Materials for

GOLM1 suppresses autophagy-mediated anti-tumor immunity in hepatocellular carcinoma

Tianqi Sui^1, 2#^, Xiaoyang Wang^2, 3#^, Lili Li^1, 2#^, Junxiao Liu^2, 4^, Nan Qiao^1, 2^, Lihua Duan^5^, Minxin Shi^6^, Jianfei Huang^7^, Heng Yang^1, 2*^, Genhong Cheng^8*^

Affiliations

^1^Center for Systems Medicine, Institute of Basic Medical Sciences, Chinese Academy of Medical Sciences & Peking Union Medical College, 100005 Beijing, China

^2^Suzhou Institute of Systems Medicine, 215123 Suzhou, Jiangsu, China

^3^Heze Vocational College, Heze Shandong，274008, China

^4^School of Life Science and Technology, China Pharmaceutical University, Nanjing, China.

^5^Department of Rheumatology and Clinical Immunology, Jiangxi Provincial People's Hospital, Nanchang, China.

^6^Affiliated Tumour Hospital of Nantong University, Nantong Tumour Hospital, 226000 Nantong, China

^7^Department of Clinical Biobank, Affiliated Hospital of Nantong University, Nantong, Jiangsu, China

^8^Department of Microbiology, Immunology & Molecular Genetics, University of California, Los Angeles, Los Angeles, CA 90095, USA

Correspondence to: Dr. Heng Yang yhmyt@hotmail.com and Dr. Genhong Cheng gcheng@mednet.ucla.edu

These authors contributed equally: Tianqi Sui, Lili Li, Xiaoyang Wang

**This PDF file includes:**

Materials and Methods

Figure S1 to S2

Tables S1 to S3

**Materials and Methods**

**Tumor tissues and clinical data**

This study recruited 261 human hepatic specimens at the Affiliated Hospital of Nantong University from March 2004 to August 2013, including HCC samples (n=161), peri-carcinomatous samples (n=48), samples of benign liver diseases (n=52). Tumor tissues collected from surgical patients were fixed with formalin and embedded with paraffin and then examined by at least two independent pathologists. All the patients had not received chemotherapy, radiotherapy and immunotherapy before their surgeries. The clinical data of all patients were collected and recorded in detail, including age, sex, HBV infection, liver cirrhosis, vascular invasion, tumor size, tumor differentiation and TNM stage. The study was conducted according to the principles of the Declaration of Helsinki and approved by the Ethical Committee of the Human Research Ethics Committee of the Affiliated Hospital of Nantong University.

**Immunohistochemistry measurement and analysis of tissue microarray**

Tissue microarray- Immunohistochemistry (TMA-IHC) was performed to measure the expression of GOLM1 protein in tissue blocks. The core tissue biopsies (2 mm in diameter) taking from individual paraffin-embedded (FFPE) sections were re-arrayed in recipient paraffin blocks by using a tissue microarray system (Quick-Ray, UT06, UNITMA, Korea). Sections from arrayed blocks were sliced into 4 *μ*m and stained with a monoclonal mouse anti-human GOLM1 antibody (dilution 1:150) (catalog TA504113, Origene) and anti-human CD8 antibody (dilution 1:200, catalog ab199016, Abcam) at 4°C overnight, followed by incubation with a biotinylated anti-mouse antibody as a secondary antibody at room temperature for 2 hours. Staining intensity was scored as follows: 0 (-, blue color, negative staining), 1 (+, yellow color, weak staining), 2 (++, orange color) moderate staining), as well as 3 (+++, brown coloraintense staining) by two pathologists. Vectra 3.0 Automated Quantitative Pathology Imaging System (PerkinElmer company，USA) was used to accurately calculate cell counts of different GOLM1 or CD8 staining intensity in specific tissues. The optimal cutoff value of GOLM1 expression was set by the X-tile software (Rimm Laboratory, Yale University; <http://www.tissuearray.org/rimmlab>). The expression levels of GOLM1 protein were graded by one two-grade scoring system, and we chose 96.5 as the cutoff point: a score of 0-96.5 was regarded as low expression， while 96.5 to 300 was regarded as high expression.

**Cell culture**

HEK293 and MCA205 cells were cultured in DMEM (catalog 11995040). H22 cells were cultured in 1640 (catalog 22400089). All of the mediums were supplemented with 10% FBS (catalog 10099141), and 100 U/mL penicillin and streptomycin (catalog 15140122) (Thermo Fisher Scientific). All cells were incubated at 37°C in a humidified air atmosphere with 5% CO_2_. For autophagy detection, RFP-GFP-LC3 lentivirus (Genechem, Shanghai) was infected in *Golm1*^-/-^ and *Golm1*^+/+^ cells and enriched with FACSAria™ III cell sorter (Becton Dickinson, San José, CA, USA).

**Construction of stable cell lines with CRISPR/Cas9 system**

SgRNA sequences target *Golm1* (forward: 5’-CACCGATTGCTAGCTCGAGAAGCG-3’; reverse: 5’-AAACCGCTTCTCGAGCTAGCAATC-3’) were synthesized and cloned into the LentiCRISPR v2 vector (a gift from Feng Zhang, plasmid #Addgene 52961). Lentivirus was produced in HEK293 cells by co-transfection of pMD2.G (plasmid # Addgene 12259), psPAX2 (plasmid # Addgene 12260) and GOLM1-sgRNA plasmids or control vector plasmid. Virus supernatant was collected at 48h post transfection, passed through a 0.45 μm filter and stored at -80 ̊C. To establish a stable knockout cell line, the lentivirus stocks were used to transduce H22 cells or MCA205 cells with 8 μg/ml polybrene. 48h post infection, cells were cultured in puromycin (2 μg/ml for H22 cells and 4 μg/ml for MCA205 cells) selection medium for at least 7 days. Monoclonal cells cultured in 96-well plate were acquired by cell sorter.

**Tumor models**

Animal experimental protocols were approved by the Institutional Animal Care and Use Committee (IACUC) of Suzhou Institute of Systems Medicine. Female C57BL/6, Balb/c, athymic nude Balb/c mice (*nu/nu*) (aged between 6–8 weeks) were purchased from Beijing Vital River Company. *Ifnar*^-/-^ C57BL/6 mice (aged between 6–8 weeks) were purchased from Model Animal Research Center of Nanjing University. Mice were randomly divided into indicated groups (5 mice/group) before inoculation. WT, negative control (transfected with CRISPR/Cas9 vector) or corresponding GOLM1 deficient tumor cells (2×10^6^ cells in 100μl PBS per mouse) were subcutaneously implanted in host mice. For orthotropic HCC mouse model, 1×10^6^ H22 cells in 100μl PBS per mouse were implanted in mouse liver. Chemotherapy was administered when tumor size was up to 40 mm^2^ (normally 7 days after tumor cell injection), by intraperitoneally (i.p.) injection of MTX (1mM, 100 μl). Tumor growth was monitored by periodic measurement using calipers displayed with multiplication of tumor length and width. Tumors were harvested on day 7-10 post implanted for flow cytometry analysis, immunofluorescence staining, ELISpot assay and RNA sequencing. Animals were sacrificed when the volume of tumors reached 350 mm^2^. Tumor progression was monitored 2-3 times per week and depicted as error bars of mean ± SEM at each time point.

**Immune cell isolation,** **flow cytometry analysis**

Syngenic solid tumors from C57BL/6 mice that injected with WT and *Golm1*^-/-^ MCA205 cells were harvested and digested into single cell suspension. Cells were strained by fluorescence-labeled antibodies against vivid yellow, CD4 (catalog 100414), CD8 (catalog100723), CD45.2 (catalog 109828), CD11b (catalog 101228), CD11c (catalog 117318), F4/80 (catalog 123116), IA/IE (catalog 107616), Ly6G (catalog 127610) and Ly6C (catalog 128026) (Biolegend). Cells were stimulated by Phorbol-12-myristate-13-acetate (PMA) (catalog ab120297), Ionomycin (catalog ab120116) and Brefeldin A (catalog ab120299) (Abcam) for 4 hours, then fixed, permeabilized and stained by IFNγ antibody (catalog 12731182, eBioscience) and detected by Attune NxT Flow Cytometer (Thermo Fisher Scientific) and analyzed with FlowJo software (Tree Star, Inc., Ashland, OR, USA).

**Tissue section and immunofluorescence staining**

Tumors were harvested at 7-10 days after subcutaneous transplantation and fixed with 4% polyformaldehyde before dehydration and embedding in OCT. For immunofluorescence staining, frozen slices were fixed in 2% polyformaldehyde. After three times washing with PBS, slides were incubated in PBS with 10% FBS to block nonspecific sites of antibody adsorption and subsequently incubated with appropriate primary antibodies: CD11b (catalog 101219, Biolegend), CD8a (catalog ab217344, Abcam), and cleaved caspase-3 (catalog 9664S, CST) at 4 ̊C overnight. Then the slices were washed three times with PBS and incubated with corresponding secondary antibody (Alexa488-conjugated goat anti-rabbit IgG, Thermo Fisher Scientific). Images were captured and processed with confocal microscope (Leica TCS SP8, installed at Suzhou Institute of Systems Medicine, China) and analyzed by ImageJ software.

**Enzyme-linked immune spot (ELISpot) assay**

IFN-γ ELISPOT assays kits were purchased from BD^TM^ Bioscience and used to detect IFN-γproducing cells in the tumor microenvironment. In detail, Multi-Screen 8 well strip Assay plate (EMD Millipore) was coated with purified anti-mouse IFNγ antibody (100 μl/well, diluted in PBS) at 4°C. After 18–24 hr co-culture with tumor single cell-suspensions (10^6^ cells per well) at 37°C in a 5% CO^2^ and humidified incubator, plate was incubated with biotinylated anti-mouse IFNγ antibody (catalog 511818KZ, BD) and streptavidin–HRP, respectively. Final substrate solution was added to stop the reaction and the plate was scanned and counted using CTL ImmunoSpot® S6 Analyzers (LLC, OH, USA) after completely dry.

**Cell death, CRT exposure,** **and ATP release quantification.**

Cells were cultured and treated with MTX (1 μM) for 24h. Early- or late- stage apoptosis was analyzed with DAPI (catalog E607303-0002, BBI Life sciences) and Annexin V (catalog 5165874X, BD Bioscience) staining following the manufacturer's instructions. CRT exposure was determined by surface staining using rabbit monoclonal antibody (catalog ab92516, Abcam), followed by staining with Alexa488-conjugated goat anti-rat IgG antibody. Extracellular ATP was quantified by ENLITEN ATP Assay System (catalog FF2000, Promega).

**Western blot**

Cells were treated as described and lysed in lysis buffer and protein concentration was determined using BCA Protein Assay Kit (catalog P0011, Beyotime). Equal amounts of total protein were separated by SDS- PAGE gels and transferred to PVDF membranes. Membranes were blocked with 5% non-fat milk in TBST. Subsequently, the membranes were incubated with specific primary antibodies: ATG101 (catalog 13492T), ATG13 (catalog 13273T), AKT (catalog 4685S), phospho-AKT (Ser473) (catalog 9271S), phospho-AKT (Thr308) (catalog 4056S), mTOR (catalog 2983T), phospho-mTOR (Ser2481) (catalog 2974T), AMPK (catalog 2532S), phospho-AMPK (catalog 2535S), ULK1 (catalog 8054T), FIP200 (catalog 12436T), PARP (catalog 9542S), FLIP (catalog 56343S), CASPASE-3 (catalog 9665S), Cleaved-CASPASE-3 (catalog 9664S), CASPASE-8 (catalog 4790S), Cleaved-CASPASE-8 (catalog 8592S), β-ACTIN (catalog 3700T) (Cell Signaling Technology), ATG7 (catalog MAB6608), ATG5 (catalog NB110-53818), LC3B (catalog NB100-2220), Beclin1 (catalog NB500-249) (Novus), and SQSTM1/p62 (catalog ab56416, Abcam). The membranes were scanned with the ChemiDoc XRS+ system (Bio-Rad, USA).

**RNA sequencing**

The grinded tissues were lysed directly and total RNA was extracted from cell lysates using RNeasy Mini Kit (catalog74104, QIAGEN). 500 ng of RNA was used for reverse transcription to cDNA with ProtoScript II Reverse Transcriptase (catalog E7420L, New England BioLabs). The double-stranded cDNA was purified with Agencourt AMPure XP Beads (catalog A63881, Beckman). Then the cDNA was ligated with the paired-end adaptors by Multiplex Oligos for RNA sequencing. Sequencing was performed with Illumina hiseq x 10 and data was analyzed with CLC Genomics Workbench 12 and Ingenuity Pathway Analysis (QIAGEN Bioinformatics).

**Statistics**

Statistical analyses were performed with software GraphPad Prism 6. The continuous variables were presented as mean ± SEM. Data with normal distribution were analyzed by one-way ANOVA or unpaired two-tailed Student’s *t* tests, and tumor growth curves were compared by the Mann-Whitney *U* test, and *P* values were indicated by * *P* < 0.05, ** *P* < 0.01 and *** *P* <0.001.

**
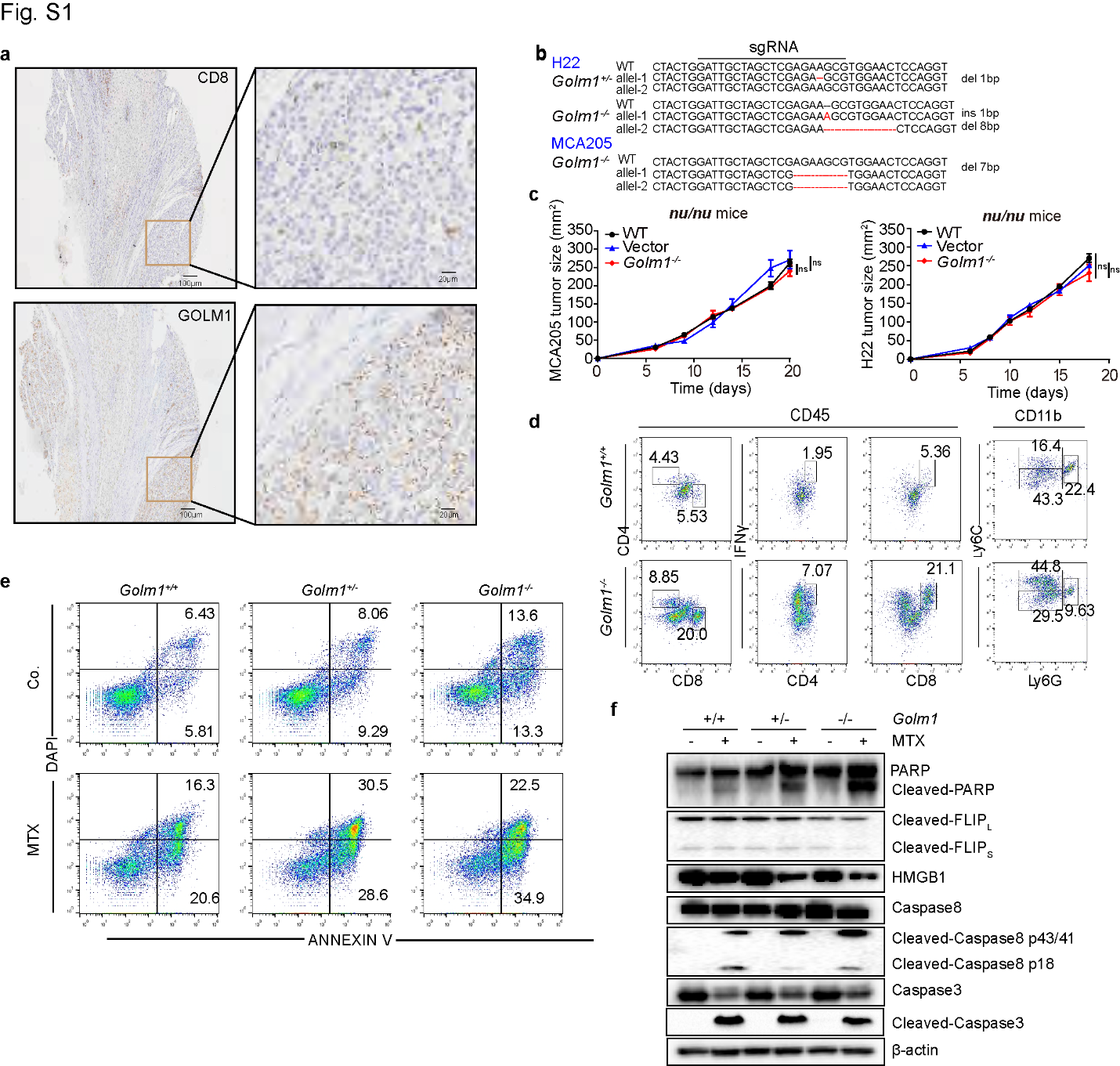
­**

**Fig. S1 a.** Expression of CD8 and GOLM1 immunohistochemical reaction in HCC malignant tissue. Tan dye indicated positive CD8 staining in the membrane of immune cells and positive GOLM1 staining in the cytoplasm of liver cells. **b.** Sequence analysis of monoclonal H22 and MCA205 cells which were presented as various inserted and deleted mutations in Golm1 coding region. **c**. Growth curves of sex- and age- match nude mice (*nu/nu*) implanted with WT, vector and *Golm1^-/-^* MCA205 or H22 cells. **d.** Representative dot plots of infiltrating immune cell populations in *Golm1^+/+^* and *Golm1^-/-^* tumors (numbers indicates the percentage of cells detected within the target gates. **e.** Cell apoptosis was detected through staining with Annexin V plus vital dye DAPI followed by flow cytometry analysis. Representative flow cytometry graphs are shown. **f.** Western blot analysis of apoptosis-related proteins in *Golm1^+/+^*, *Golm1^+/-^* and *Golm1^-/-^* H22 cells. The quantitative variables between two groups are analyzed by the Mann-Whitney U test (b) and unpaired Student`s *t* test (f). Quantitative data are represented as mean ± SEM; ns, not significant.

**
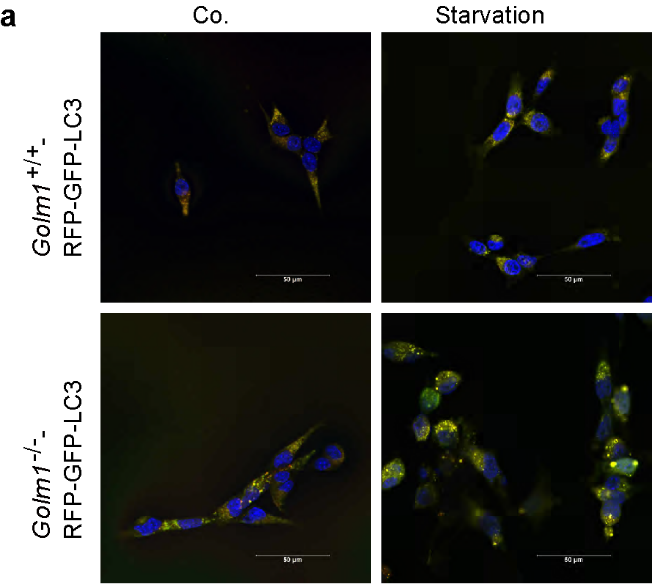
**

**Fig.S2 a.** *Golm1^+/+^* and *Golm1^-/-^* MCA205 cell line stably expressing RFP-GFP-LC3 reporter protein were generated via lentivirus-mediated overexpression. The cells that exhibited a large number of RFP-GFP-LC3 dots after treatment of EBSS (3 hours) were analyzed by confocal immunofluorescence. Representative merged images were shown. Cell nuclei is in blue, and bars=50 μm.

**
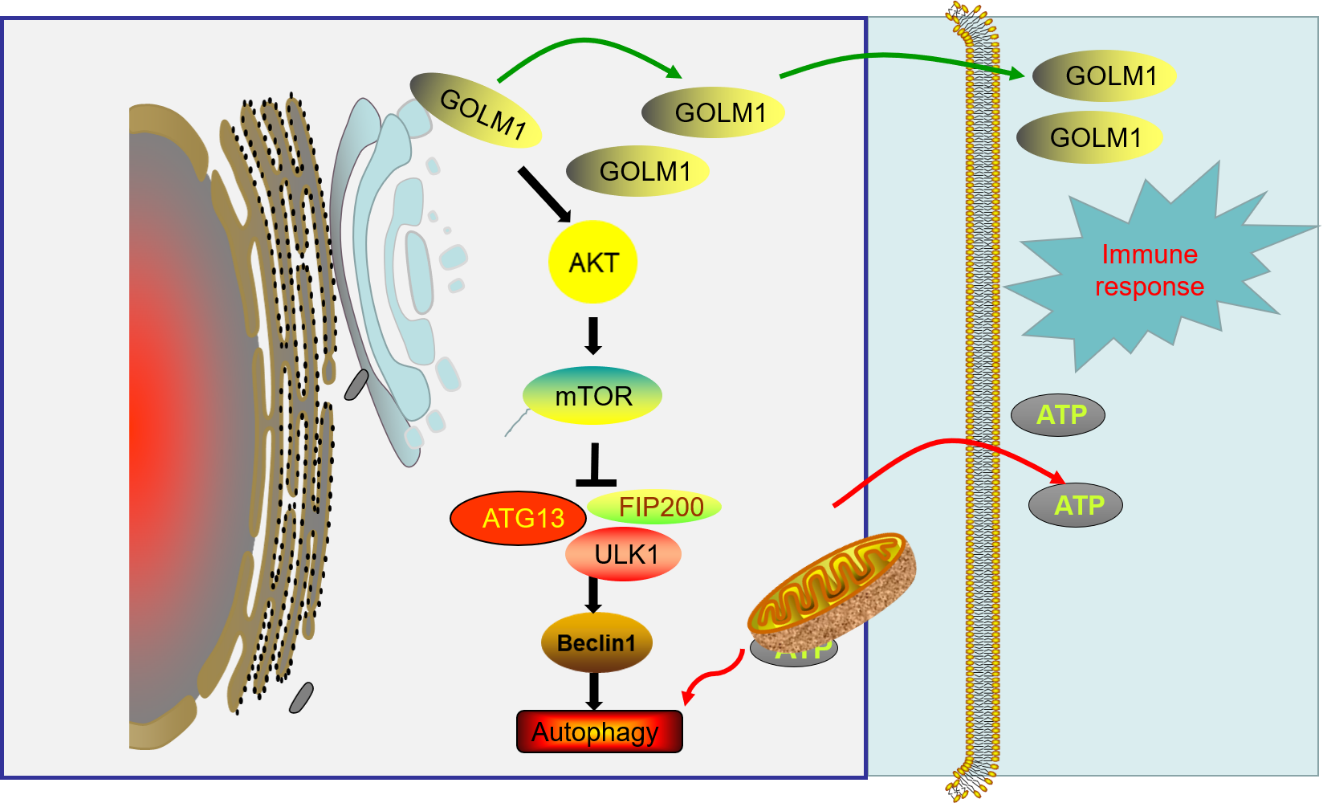
**

**Fig.S3** The proposed work model of Golm1 regulation anti-tumor immunity.

**Table 1**

Clinical demographic and pathological characteristics of the HCC patients in groups of different GOLM1 expression.

| **Parameters** | **Low or no GOLM1 expression (n=74)** | **High expression GOLM1 (n=87)** |
| --- | --- | --- |
| Ages, years | 53.11±10.85 | 52.24±8.94 |
| Sex, n (%)  Male  Female | 56 (75.68)  18 (24.32) | 65 (74.71)  22 (25.29) |
| Viral cause, n (%)  HBV  Non HBV infection | 48 (64.86)  26 (35.14) | 65 (74.71)  22 (25.29) |
| Liver cirrhosis, n (%)  Present  Absent | 44 (59.50)  30(40.50) | 56 (64.37)  31 (35.63)) |
| Vascular invasion, n (%)  Invasion  Not invasion | 22 (29.73)  52 (70.27) | 38 (43.68)  49 (56.32) |
| Tumor size, n (%)  >=5 cm  <5 cm  missing | 21 (28.38)  49 (66.22)  4 (5.41) | 34 (39.08)  46 (52.97)  7 (8.05) |
| Tumor differentiation, n (%)  High differentiation  Middle differentiation  Low differentiation  Missing | 18 (24.32)  39 (52.70)  12 (16.22)  5 (6.76) | 6 (6.90)  56 (64.36)  18 (20.69)  7(8.05) |
| TNM stage, n (%)  Ⅰ  Ⅱ  Ⅲ  Ⅳ  Missing | 35 (47.30)  24 (32.43)  4 (5.41)  1 (1.35)  10 (13.51) | 33 (37.93)  37 (42.52)  6(6.90)  1 (1.15)  10 (11.50) |

**Table 2**

GOLM1 and CD8 protein levels in liver tissues between GOLM1 low- and high-expression groups (cut-off value=96.5).

| **Parameters (IHC score)** | **Low or no expression** | **High expression** | ***P* value** |
| --- | --- | --- | --- |
| GOLM1 | 83.25±34.98 | 171.35±24.38 | <0.001 |
| CD8 | 140.23±49.23 | 123.70±46.40 | 0.0351 |

**Table 3**

**Comparison GOLM1 levels according to clinical features of HCC patients.**

| **Parameters** | **HCC patients (n=161)** | **GOLM1** | |
| --- | --- | --- | --- |
|  |  | **Mean±SD** | ***P* value** |
| Viral cause, n (%)  HBV infection  Non HBV infection | 113 (70.19)  48 (29.81) | 126.04±55.85  132.90±51.98 | 0.4551 |
| Liver cirrhosis, n (%)  Present  Absent | 100 (62.11)  61 (37.89) | 127.99±57.45  132.60±50.45 | 0.4387 |
| Vascular invasion, n (%)  Invasion  Not invasion  Missing | 61 (37.89)  92 (57.14)  8 (4.97) | 125.21±54.35  138.01±51.23 | 0.0666 |
| Tumor size, n (%)  >=5 cm  <5 cm  Missing | 55 (34.16)  95 (59.01)  11 (6.83) | 136.05±53.01  125.85±53.65 | 0.4087 |
| Tumor differentiation, n (%)  High differentiation  Middle differentiation  Low differentiation  Missing | 24 (14.91)  95 (59.01)  30 (18.63)  12 (7.45) | 97.31±48.52  137.74±52.72  139.57±44.96 | 0.0009 |
| TNM stage, n (%)  Ⅰ  Ⅱ  Ⅲ  Ⅳ  Missing | 68 (42.24)  61 (37.89)  10 (6.21)  2 (1.24)  20 (12.42) | 125.16±51.69  134.84±53.96  133.205±61.54  149.00±101.82 | 0.4187 |
